# μ-Oxo-bridged diiron(iii) complexes of tripodal 4N ligands as catalysts for alkane hydroxylation reaction using *m*-CPBA as an oxidant: substrate *vs.* self hydroxylation†

**DOI:** 10.1039/d1ra03135j

**Published:** 2021-06-17

**Authors:** Mani Balamurugan, Eringathodi Suresh, Mallayan Palaniandavar

**Affiliations:** School of Chemistry, Bharathidasan University Tiruchirappalli 620 024 Tamil Nadu India palanim51@yahoo.com palaniandavarm@gmail.com; Analytical Science Discipline, Central Salt and Marine Chemicals Research Institute Bhavnagar 364 002 India

## Abstract

A series of non-heme μ-oxo-bridged dinuclear iron(iii) complexes of the type [Fe_2_(μ-O)(L1–L6)_2_Cl_2_]Cl_2_1–6 have been isolated and their catalytic activity towards oxidative transformation of alkanes into alcohols has been studied using *m*-choloroperbenzoic acid (*m*-CPBA) as an oxidant. All the complexes were characterized by CHN, electrochemical, and UV-visible spectroscopic techniques. The molecular structures of 2 and 5 have been determined successfully by single crystal X-ray diffraction analysis and both possesses octahedral coordination geometry and each iron atom is coordinated by four nitrogen atoms of the 4N ligand and a bridging oxygen. The sixth position of each octahedron is coordinated by a chloride ion. The (μ-oxo)diiron(iii) core is linear in 2 (Fe–O–Fe, 180.0°), whereas it is non-linear (Fe–O–Fe, 161°) in 5. All the diiron(iii) complexes show quasi-reversible one electron transfer in the cyclic voltammagram and catalyze the hydroxylation of alkanes like cyclohexane, adamantane with *m*-CPBA as an oxidant. In acetonitrile solution, adding excess *m*-CPBA to the diiron(iii) complex 2 without chloride ions leads to intramolecular hydroxylation reaction of the oxidant. Interestingly, 2 catalyzes alkane hydroxylation in the presence of chloride ions, but intramolecular hydroxylation in the absence of chloride ions. The observed selectivity for cyclohexane (A/K, 5–7) and adamantane (3°/2°, 9–18) suggests the involvement of high-valent iron–oxo species rather than freely diffusing radicals in the catalytic reaction. Moreover, 4 oxidizes (A/K, 7) cyclohexane very efficiently up to 513 TON while 5 oxidizes adamantane with good selectivity (3°/2°, 18) using *m*-CPBA as an oxidant. The electronic effects of ligand donors dictate the efficiency and selectivity of catalytic hydroxylation of alkanes.

## Abstract 2

The ligand stereoelectronic effect of diiron(iii) complexes determines the efficiency and selectivity of catalytic alkane hydroxylation with *m*-CPBA as an oxidant.

## Graphical abstract

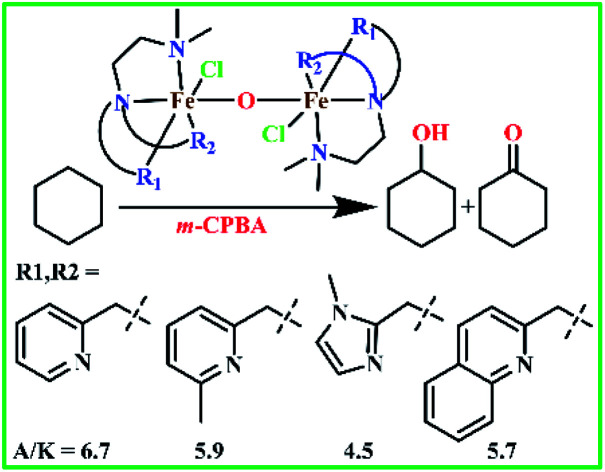

## Introduction

**Scheme 1:**
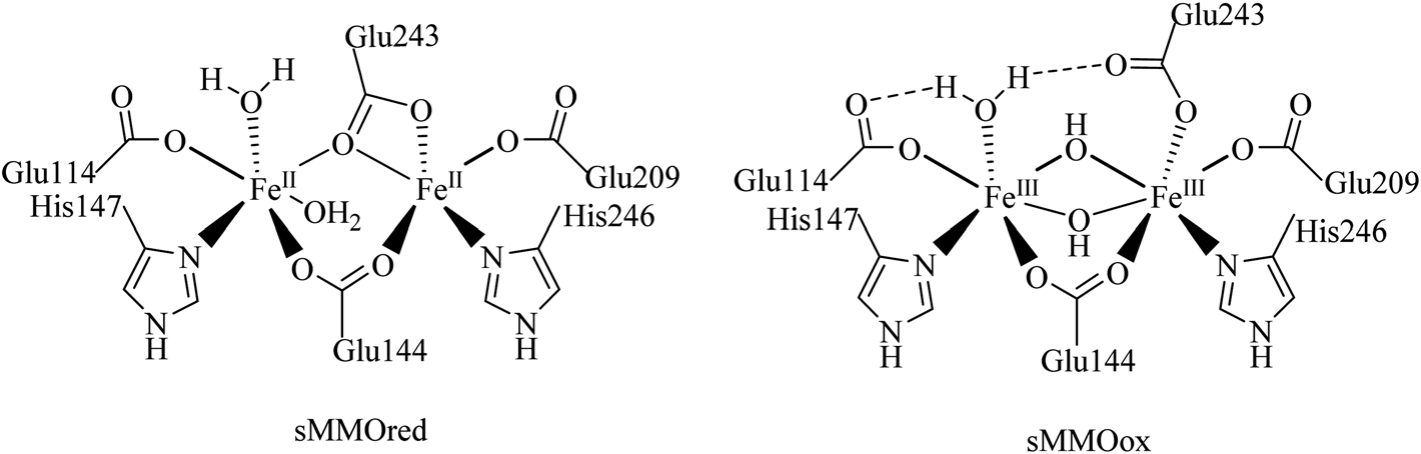
Active site structures of soluble methane monooxygenases.

In nature, non-heme diiron enzymes, such as methane monooxygenases, ribonucleotide reductases *etc.*, activate oxygen and catalyze alkane oxidation reactions. Among these enzymes, soluble methane monooxygenases having a μ-oxo bridged diiron core are the widely investigated metalloenzymes involved in the conversion of methane into methanol using molecular oxygen under ambient conditions.^1–5^ Therefore, the diiron(iii) complexes having an Fe–O–Fe core have received greater attention in the field of hydrocarbon oxidation under mild conditions (Scheme 1).^6–8^ Significantly, nature has evolved a wide variety of coordination environments around iron centers to differentiate the function of the enzymes from one another and utilised distinct intermediates, which are supposed to be involved in their intrinsic catalytic behaviour.^9–13^ In the case of heme enzymes the oxoiron(iv) porphyrin π–cation radical is found to be the oxidizing intermediate involved in alkane hydroxylation.^14,15^ On the other hand, the involvement of the Fe^IV^_2_O_2_ diamond core is observed as the reactive intermediate species in methane oxidation by the soluble methane monooxygenases (sMMO) and the enzymes hold two oxidizing equivalents divided on two iron centers.^13,16^ As alkane functionalization is an important chemical transformation in the field of organic and synthetic chemistry, selective oxidation of hydrocarbons under mild conditions has become an exciting and challenging scientific objective. Therefore, the development of a diiron catalyst for alkane hydroxylation reaction has attracted greater attention to illustrate the oxidizing intermediates and catalytic pathway of enzymes.^17–19^

In earlier studies attempts have been made to reproduce the structural and functional aspects of the enzymes and several model complexes have been reported as both functional and structural models for methane monooxygenases enzymes.^2,20–35^ A few μ-oxo-bridged diiron(ii) complexes were developed as structural mimics of the active center of sMMO and related enzymes, in which the active site coordination environment of the sMMO have been mimicked.^2,36–41^ Also, the involvement of high valent Fe^IV^=O species in the alkane hydroxylation reaction was proved and are characterized the species by X-ray crystallographic techniques.^2,42–46^ The diiron(iii) complexes of tris(2-pyridylmethyl)amine (TPA) and related ligands are known as effective sMMO models.^47^ However, such ligands do not stabilize the diiron core in solution, and the resulting complexes display varied reactivity, depending on them being mono- or diiron complexes.^48,51,52^ Whereas, the sMMO model derived from TPA-containing dinucleating ligand has been stabilized diiron core in solution and act as effective catalyst for alkane functionalization.^49^

**Scheme 2:**
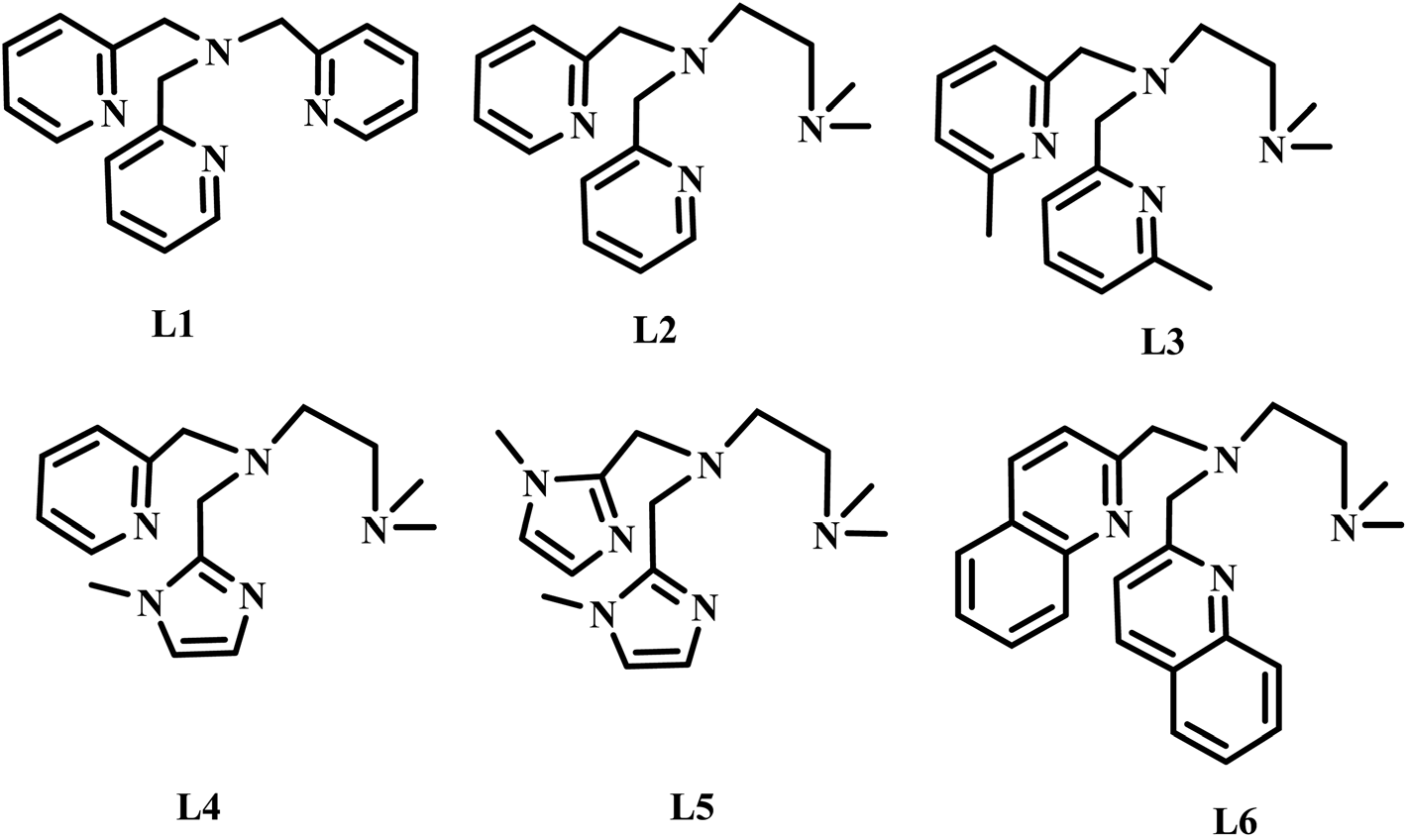
Structures of 4N ligands employed in the study.

The diiron(iii) complexes have been utilised as catalysts for various alkane oxidation reactions using different types of oxidants such as molecular oxygen, hydrogen peroxide (H_2_O_2_), *t*-butyl hydroperoxide (*t*-BuOOH) and *m*-chloroperbenzoic acid (*m*-CPBA). For instance, the unsymmetrical diiron-μ-oxo complex [L^3^_4_Fe^III^(Cl)(μ-O)Fe^III^Cl_3_], where L^3^_4_ is *N*,*N*′-dimethyl-*N*,*N*′-bis(2-pyridylmethyl)propane-1,3-diamine, exhibits hexane oxidation reaction with molecular oxygen as oxidant in the presence of trimethylhydroquinone as reductant.^50^ Various diiron(iii) complexes with pyridyl, imidazolyl and benzimidazolyl nitrogen donating ligands have been used as catalysts for alkane and benzene oxidation reactions using H_2_O_2_ or *t*-BuOOH or *m*-CPBA as oxidants and achieved moderate to good selectivity.^51–57^ Similarly, various diiron(iii) complexes with phenolate ligands have been used as catalysts for alkane oxidation reactions with good alcohol selectivity.^55,58–60^ Interestingly, various diiron(iii) complexes with carboxylate oxygen as ligand donors exhibited efficient and selective oxidation of alkanes with various oxidants and with high A/K ratio.^61–65^ Likewise, the diiron(iii) complexes with N-heterocyclic carbene ligands catalyzed the benzene hydroxylation to phenol with H_2_O_2_ as oxidant.^66^ Interestingly, several diiron(iii) complexes catalyzes intra-molecular aliphatic^67^ and aromatic oxidation reactions, where the phenyl group is usually oxidized using various oxidants.^68–72^ Although, various μ-oxo-bridged non-heme diiron(iii) complexes that mimic the functions of diiron enzymes have been reported earlier, the design and study of diiron(iii) complexes would enhance the understanding further to utilize the complexes as excellent catalysts for the oxidation of organic substrates, particularly for alkane functionalization and alkene epoxidation reactions. Moreover, the factors determining the selectivity as well as efficiency of the catalysts remain still unclear. Even though, several studies proved the involvement of Fe^IV^=O species in alkane hydroxylation, it is difficult to eliminate the possibility of involvement of Fe^V^=O species and a few reports support the involvement of later species also in alkane hydroxylation reaction.^73–75^ All the above observations prompted us to isolate a few diiron(iii) complexes of systematically varied tripodal 4N ligands having pyridine, imidazole and sterically demanding quinoline moieties and weakly binding –NMe_2_ groups and to study the ligand stereoelectronic factors upon the efficiency as well as alcohol product selectivity of the complexes as catalysts for alkane hydroxylation reaction (Scheme 2). All the present diiron(iii) complexes catalyse the hydroxylation of alkanes like cyclohexane and adamantane efficiently with good alcohol selectivity using *m*-CPBA as the oxidant within an hour. Further, when the pyridine moiety in the diiron(iii) catalyst is replaced with –NMe_2_ donor group the selectivity of the catalyst remains approximately the same. In contrast, for adamantane oxidation the incorporation of sterically hindering quinolyl donor around diiron(iii) leads to a high 3°/2° bond selectivity.

## Experimental section

### Materials

*N*,*N*-Dimethylethylenediamine, 2-picolylamine, pyridine-2-carboxaldehyde, 1-methylimidazole-carboxaldehyde, sodium triacetoxyborohydride, adamantane, silver perchlorate monohydrate, 3-chlorosalicylic acid (Aldrich), 6-methylpyridine-2-carboxaldehyde and quinolin-2-carboxaldehyde (Alfa Aesar), tetraethylammonium chloride (Lancaster), cyclohexane (Ranbaxy, India), iron(iii) chloride hexahydrate (Loba, India), acetic acid, pentane (Merck, India) were used as received. The supporting electrolyte tetrabutylammonium perchlorate (NBu_4_ClO_4_, G. F. Smith, USA) was recrystallized twice from aqueous ethanol. Diethyl ether and tetrahydrofuran were dried over sodium metal with benzophenone indicator. Acetonitrile, dichloromethane (Merck, India) and methanol (Fisher Scientific, India) were distilled before use.

### Synthesis of ligands

#### Tris(2-pyridylmethyl)amine (tpa) (L1)

This ligand was prepared as reported^76^ elsewhere. The ^1^H NMR data agreed well with the reported one.

#### *N*,*N*-Dimethyl-*N*′,*N*′-bis(pyrid-2-ylmethyl)ethane-1,2-diamine (L2)

The ligand was prepared as reported^76^ elsewhere. Yield: 1.19 g (88%). ^1^H NMR (400 MHz, CDCl_3_): *δ* 2.18 (s, 6H), 2.41 (t, 2H), 2.65 (t, 2H), 3.87 (s, 4H), 7.15 (t, 2H), 7.53 (d, 2H), 7.65 (t, 2H), 8.51 (d, 2H). EI-MS *m*/*z* = 270.1 C_16_H_22_N_4_˙^+^.

#### *N*,*N*-Dimethyl-*N*′,*N*′-bis(6-methylpyrid-2-ylmethyl)ethane-1,2-diamine (L3)

The ligand was prepared by employing the method used for synthesizing L2 and using 6-methylpyridine-2-carboxaldehyde instead of pyridine-2-carboxaldehyde. Yield: 1.16 g (79%). ^1^H NMR (400 MHz, CDCl_3_): *δ* 2.14 (s, 6H, N–Me_2_), 2.42 (t, 2H, –CH_2_–NMe_2_), 2.60 (t, 2H, –CH_2_–CH_2_–NMe_2_), 3.74 (s, 4H, –CH_2_–Py), 7.15 (t, 1H, –5H–Py), 7.53 (d, 1H, –3H–Py), 7.65 (t, 1H, –4H–Py), 2.35 (s, 6H, Py–Me).

#### *N*,*N-*Dimethyl*-N*′-(1-methyl-1*H*-imidazol-2-ylmethyl)-*N*′-(pyrid-2-ylmethyl)ethane-1,2-diamine (L4)

The ligand was prepared as reported^77^ elsewhere. Yield: 1.12g (82%). ^1^H NMR (400 MHz, CDCl_3_): *δ* 2.13 (s, 6H), 2.44 (t, 2H), 2.63 (t, 2H), 3.67 (s, 3H), 3.58 (s, 2H), 3.78 (s, 2H), 6.78 (s, 1H), 6.86 (s, 1H), 7.17 (t, 1H), 7.46 (d, 1H), 7.63 (t, 1H), 8.58 (d, 1H). EI-MS *m*/*z* = 273.2 C_15_H_23_N_5_˙^+^.

#### *N*,*N-*Dimethyl*-N*′,*N*′-bis(1-methyl-1*H*-imidazol-2-ylmethyl)ethane-1,2-diamine (L5)

The ligand was prepared as reported^77^ elsewhere. Yield: 1.05 g (76%). ^1^H NMR (400 MHz, CDCl_3_): *δ* 2.14 (s, 6H), 2.41 (t, 2H), 2.59 (t, 2H), 3.68 (s, 6H), 3.59 (s, 4H), 6.80 (s, 2H), 6.87 (s, 2H). EI-MS *m*/*z* = 276.2 C_14_H_24_N_6_˙^+^.

#### *N*,*N-*Dimethyl*-N*′,*N*′-bis(quinolin-2-ylmethyl)ethane-1,2-diamine (L6)

The ligand was prepared as reported^77^ elsewhere. The brown oil was used without further purification for the isolation of the complex. Yield: 1.28 g (69%). ^1^H NMR (400 MHz, CDCl_3_): *δ* 2.13 (s, 6H), 2.42 (t, 2H), 2.62 (t, 2H), 3.80 (s, 4H), 7.15 (d, 2H), 7.42 (t, 2H), 7.57 (t, 2H), 7.67 (d, 2H), 7.89 (d, 2H), 8.13 (d, 2H). EI-MS *m*/*z* = 370.2 C_24_H_26_N_4_˙^+^.

### Synthesis of complexes

#### [Fe_2_(μ-O)(L1)_2_Cl_2_]Cl_2_1

The complex was prepared as reported elseware.^47^ Yield: 0.32 g (74%). Anal. calcd. for C_36_H_36_Cl_4_Fe_2_N_8_O: C, 50.86; H, 4.27; N, 13.18. Found: C, 50.78 H, 4.31; N, 13.13.

#### [Fe_2_(μ-O)(L2)_2_Cl_2_]Cl_2_·MeOH·H_2_O 2

A methanol solution (5 mL) of (Et_4_N)_2_[Fe_2_OCl_6_] (300 mg, 0.5 mmol) was added to a methanol solution (5 mL) of L2 (135.1 mg, 0.5 mmol) with stirring at room temperature. After stirring for 1 h the solution was slowly evaporated leading to the formation of red colored crystals suitable for X-ray diffraction studies. Yield: 0.275 g (68%). Anal. calcd. for C_32_H_44_Cl_4_Fe_2_N_8_O: C, 47.44; H, 5.47; N, 13.83. Found: C, 47.50; H, 5.40; N, 13.88.

#### [Fe_2_(μ-O)(L3)_2_Cl_2_]Cl_2_3

The procedure used for synthesis of 1 was used for the synthesis of 3. Yield: 0.28 g (65%). Anal. calcd. for C_36_H_52_Cl_4_Fe_2_N_8_O: C, 49.91; H, 6.05; N, 12.93. Found: C, 49.93; H, 6.11; N, 12.97.

#### [Fe_2_(μ-O)(L4)_2_Cl_2_]Cl_2_4

The procedure employed for synthesis of 1 was used for synthesis of 4. After stirring for 1 h the brown red precipitate was filtered off and washed with acetone. Yield: 0.29 g (71%). Anal. calcd. for C_30_H_46_Cl_4_Fe_2_N_8_O: C, 44.14; H, 5.68; N, 17.16. Found: C, 44.20; H, 5.75; N, 17.12.

#### [Fe_2_(μ-O)(L5)_2_Cl_2_]Cl_2_5

The procedure employed for synthesis of 1 was used for synthesis of 5. After stirring for 1 h the brown red precipitate was filtered off and washed with acetone. Yield: 0.30 g (73%). Anal. calcd. for C_28_H_48_Cl_4_Fe_2_N_12_O: C, 40.90; H, 5.88; N, 20.44. Found: C, 40.95; H, 5.81; N, 20.37.

#### [Fe_2_(μ-O)(L6)_2_Cl_2_]Cl_2_6

The procedure employed for synthesis of 1 was used for synthesis of 6. After stirring for 1 h the brown red precipitate was filtered off and washed with acetone. Yield: 0.29 g (58%). Anal. calcd. for C_48_H_52_Cl_4_Fe_2_N_8_O: C, 57.05; H, 5.19; N, 11.09. Found: C, 57.11; H, 5.17; N, 11.07.

### Catalytic oxidations

The oxidation of alkanes was carried out at room temperature under research grade nitrogen atmosphere. In a typical reaction, oxidant *m*-CPBA (0.8 mol dm^−3^) was added to the mixture of diiron(iii) complex (1 × 10^−3^ mmol dm^−3^) and alkanes (3 mol dm^−3^) and in CH_2_Cl_2_ : CH_3_CN mixture (4 : 1 v/v). After 30 min the reaction mixture was quenched with triphenylphosphine, the reaction mixture was filtered over a silica column and then eluted with diethylether. An internal standard (bromobenzene) was added at this point and the solution was subjected to GC analysis. The mixture of organic products were identified by Agilent GC-MS and quantitatively analyzed by HP 6890 series GC equipped with HP-5 capillary column (30 m × 0.32 mm × 2.5 μm) using a calibration curve obtained with authentic compounds. All of the products were quantified using GC (FID) with the following temperature program: injector temperature 130 °C; initial temperature 60 °C, heating rate 10 °C min^−1^ to 130 °C, increasing the temperature to 160 °C at a rate of 2 °C min^−1^, and then increasing the temperature to 260 °C at a rate of 5 °C min^−1^; FID temperature 280 °C. GC-MS analysis was performed under conditions identical to those used for GC analysis. The averages of three measurements are reported.

### Physical measurements

Elemental analyses were performed on a Perkin Elmer Series II CHNS/O Analyzer 2400. ^1^H NMR spectra were recorded on a Bruker 400 MHz NMR spectrometer. Electronic spectra were recorded on Agilent 8453 Diode Array Spectrophotometer. Low temperature spectra were obtained on Agilent 8453 Diode Array Spectrophotometer equipped with an UNISOKU USP-203 cryostat. ESI-MS analyses were recorded on a Micromass Quattro II triple quadrupole mass spectrometer. Cyclic voltammetry (CV) and differential pulse voltammetry (DPV) were performed at 25 ± 0.2 °C using a three-electrode cell configuration. A platinum sphere, a platinum plate and Ag(s)/AgNO_3_ were used as working, auxiliary and reference electrodes, respectively. The platinum sphere electrode was sonicated for two minutes in dilute nitric acid, dilute hydrazine hydrate and in double distilled water to remove the impurities. The reference electrode for non-aqueous solution was Ag(s)/Ag^+^, which consists of a Ag wire immersed in a solution of AgNO_3_ (0.01 M) and tetra-N-butylammonium perchlorate (0.1 M) in acetonitrile placed in a tube fitted with a Vycor plug. The instruments utilized included an EG & G PAR 273 Potentiostat/Galvanostat and P-IV computer along with EG & G M270 software to carry out the experiments and to acquire the data. The temperature of the electrochemical cell was maintained by a cryo-circulator (HAAKE D8-G). The *E*_1/2_ observed under identical conditions for Fc/Fc^+^ couple in acetonitrile was 0.102 V with respect to the Ag/Ag^+^ reference electrode. The experimental solutions were deoxygenated by bubbling research grade nitrogen and an atmosphere of nitrogen was maintained over the solution during measurements. The products were analyzed by using Hewlett Packard (HP) 6890 GC series Gas Chromatograph equipped with a FID detector and a HP-5 capillary column (30 m × 0.32 mm × 2.5 μm). GC-MS analysis was performed on an Agilent GC-MS equipped with 7890A GC series (HP-5 capillary column) and 5975C inert MSD under conditions that are identical to that used for GC analysis.

### Crystal data collection and structure refinement

The diffraction experiments were carried out on a Bruker SMART APEX diffractometer equipped with a CCD area detector. High quality crystals, suitable for X-ray diffraction was chosen after careful examination under an optical microscope. Intensity data for the crystal was collected using MoK_α_ (*λ* = 0.71073 Å) radiation on a Bruker SMART APEX diffractometer equipped with CCD area detector at 100 and 293 K. The data integration and reduction was processed with SAINT software. An empirical absorption correction was applied to the collected reflections with SADABS. The structure was solved by direct methods using SHELXTL and refined on *F*^2^ by the full-matrix least-squares technique using the SHELXL-97 package.^78–80^ Even though, the data of 2 was collected at LN temperature (100 K) during the structure solution it was observed that carbon atoms of the coordinated acetonitrile molecule in 2 appeared as diffused peaks and the methyl carbon is disordered. Both these carbon atoms were located from the difference Fourier map and since the peak heights of the carbon atoms were small and diffused the whole coordinated CH_3_CN molecule was refined only isotropically. For the disordered methyl carbon, the occupancy factor is assigned using FVAR command. Crystal data and additional details of the data collection and refinement of the structure are presented in Table 1. The selected bond lengths and bond angles are listed in Table 2.

**Table 1:** Crystal data and structure refinement details for [Fe_2_O(L2)_2_(Cl)_2_]Cl_2_·CH_3_OH·H_2_O 2 and [Fe_2_O(L5)_2_(Cl)_2_]Cl_2_5

	2	5
Empirical formula	C_33_H_50_Cl_4_Fe_2_N_8_O_3_	C_28_H_63_Cl_4_Fe_2_N_12_O
Formula weight/g mol^−1^	860.31	837.40
Crystal habit, colour	Blocks, red	Blocks, red
Crystal system	Orthorhombic	Monoclinic
Crystal size	0.23 × 0.33 × 0.56 mm	0.22 × 0.36 × 0.48 mm
Space group	*Pccn*	*P*2_1_/*n*
*a*, Å	17.8183(11)	17.8072(5)
*b*, Å	23.7713(15)	15.7838(5)
*c*, Å	9.3271(6)	17.8744(5)
*α*, deg	90.00	90.00
*β*, deg	90.00	116.3110(10)
*γ*, deg	90.00	90.00
*V*/Å^3^	3950.6(4)	4503.4(2)
*Z*	4	4
*ρ*_calcd_/g cm^−3^	1.446	1.235
*F*(000)	1792	1772
*T*/K	273	296
No. of reflections collected	22 432	12 463
No. of unique reflections	4698	8536
Radiation (MoKα)/Å	0.71073	0.71073
Goodness-of-fit on *F*^2^	1.115	1.018
Number of refined parameters	257	510
*R*_1_/w*R*_2_ [*I* > 2σ(*I*)]^a^	0.0752/0.1997	0.0806/0.2421
*R*_1_/w*R*_2_ (all data)	0.0824/0.2071	0.1131/0.2771

a *R*_1_ = [Σ(||*F*_o_| − |*F*_c_||)/Σ|*F*_o_|]; w*R*_2_ = {[Σ(*w*(*F*_o_^2^ − *F*_c_^2^)^2^)/Σ(*wF*_o_^4^)]^1/2^}.

**Table 2:** Selected bond lengths [Å] and bond angles [°] for 2 and 5

2		5	
**Bond lengths/Å**			
Fe(1)–N(1)	2.138(3)	Fe(1)–N(1)	2.099(4)
Fe(1)–N(2)	2.219(3)	Fe(1)–N(3)	2.367(4)
Fe(1)–N(3)	2.129(4)	Fe(1)–N(4)	2.095(4)
Fe(1)–N(4)	2.369(4)	Fe(1)–N(6)	2.277(4)
Fe(1)–O(1)	1.7959(6)	Fe(1)–O(1)	1.791(4)
Fe(1)–Cl(1)	2.2996(11)	Fe(1)–Cl(2)	2.4108(15)
		Fe(2)–N(7)	2.118(5)
		Fe(2)–N(9)	2.335(4)
		Fe(2)–N(10)	2.109(4)
		Fe(2)–N(12)	2.266(5)
		Fe(2)–O(1)	1.778(3)
		Fe(2)–Cl(2)	2.3871(15)

**Bond angles/°**			
N(1)–Fe(1)–N(2)	77.13(13)	O(1)–Fe(1)–N(4)	107.04(16)
N(1)–Fe(1)–N(3)	154.13(14)	O(1)–Fe(1)–N(1)	104.37(17)
N(1)–Fe(1)–N(4)	86.66(15)	N(4)–Fe(1)–N(1)	148.57(17)
N(2)–Fe(1)–N(3)	77.00(13)	O(1)–Fe(1)–N(6)	95.06(17)
N(2)–Fe(1)–N(4)	77.98(13)	N(4)–Fe(1)–N(6)	88.78(17)
N(3)–Fe(1)–N(4)	88.27(15)	N(1)–Fe(1)–N(6)	87.47(17)
O(1)–Fe(1)–N(1)	89.90(9)	O(1)–Fe(1)–N(3)	172.24(17)
O(1)–Fe(1)–N(2)	93.93(9)	N(4)–Fe(1)–N(3)	73.92(15)
O(1)–Fe(1)–N(3)	91.58(10)	N(1)–Fe(1)–N(3)	74.82(16)
O(1)–Fe(1)–N(4)	171.73(10)	N(6)–Fe(1)–N(3)	77.22(16)
Cl(1)–Fe(1)–O(1)	100.87(4)	O(1)–Fe(1)–Cl(1)	99.78(13)
Cl(1)–Fe(1)–N(1)	101.77(10)	N(4)–Fe(1)–Cl(1)	87.58(13)
Cl(1)–Fe(1)–N(2)	165.17(10)	N(1)–Fe(1)–Cl(1)	88.14(13)
Cl(1)–Fe(1)–N(3)	103.29(10)	N(6)–Fe(1)–Cl(1)	165.15(13)
Cl(1)–Fe(1)–N(4)	87.20(10)	N(3)–Fe(1)–Cl(1)	87.93(12)
Fe(1)–O(1)–Fe(1_a)	180.00	O(1)–Fe(2)–N(7)	104.49(19)
		O(1)–Fe(2)–N(10)	106.50(17)
		N(7)–Fe(2)–N(10)	148.97(19)
		O(1)–Fe(2)–N(12)	94.76(18)
		N(7)–Fe(2)–N(12)	91.2(2)
		N(10)–Fe(2)–N(12)	84.8(2)
		O(1)–Fe(2)–N(9)	173.48(16)
		N(7)–Fe(2)–N(9)	73.88(19)
		N(10)–Fe(2)–N(9)	75.15(16)
		N(12)–Fe(2)–N(9)	79.04(17)
		O(1)–Fe(2)–Cl(2)	99.52(13)
		N(7)–Fe(2)–Cl(2)	90.24(15)
		N(10)–Fe(2)–Cl(2)	86.17(14)
		N(12)–Fe(2)–Cl(2)	164.80(15)
		N(9)–Fe(2)–Cl(2)	86.84(12)
		Fe(2)–O(1)–Fe(1)	161.1(2)

## Results and discussion

### Syntheses and characterization of ligands and their diiron(iii) complexes

The tripodal tetradentate 4N ligands L1–L6 (Scheme 1) were synthesized according to known procedures which involve reductive amination reaction. The ligands L1–L6 were prepared by reductive amination of 2-picolylamine with two moles of pyridine-2-carboxaldehyde (L1) and *N*,*N*-di-methylethylenediamine with two moles of pyridine-2-carboxaldehyde (L2) or 6-methylpyridine-2-carboxaldehyde (L3) or 6-bromopyridine-2-carboxaldehyde (L4) or 1-methylimidazole-2-carboxaldehyde (L5) or quinoline-2-carboxaldehyde (L6) using sodium triacetoxyborohydride as reducing agent and were characterized by ^1^H NMR spectroscopy and mass spectrometry. The reaction of (Et_4_N)_2_[FeOCl_6_] in acetone with the 4N ligands results in the immediate formation of the reddish brown precipitate corresponds to the diiron(iii) complexes of the type [Fe_2_O(L)Cl_2_]Cl_2_. All the complexes were characterized by using elemental analysis, electronic spectroscopy and electrochemical methods. The formulation of the complexes based on elemental analysis was confirmed by determining the X-ray crystal structure of 2. Also, the ESI-MS analysis of all the complexes in methanol/acetonitrile mixture reveal the existence of the Fe–O–Fe structural motif in solution corresponding to the [Fe_2_(μ-O)(L)_2_Cl_2_] core. The asymmetric Fe–O stretching vibration observed around 835 cm^−1^ in the IR spectra (KBr) of all the complexes are consistent with the presence of the Fe–O–Fe core observed in their X-ray structures. All the diiron(iii) complexes were employed as catalysts for alkane hydroxylation using *m*-CPBA as oxidant. The tripodal ligands with different electron-releasing abilities are expected to play an important role in determining the stability of the intermediate involved in the catalytic cycle and hence the reactivity.

### Description of the X-ray crystal structures of [Fe_2_(μ-O)(L2)_2_Cl_2_]Cl_2_·MeOH 1 and [Fe_2_(μ-O)(L5)_2_Cl_2_]Cl_2_5

**Fig. 1:**
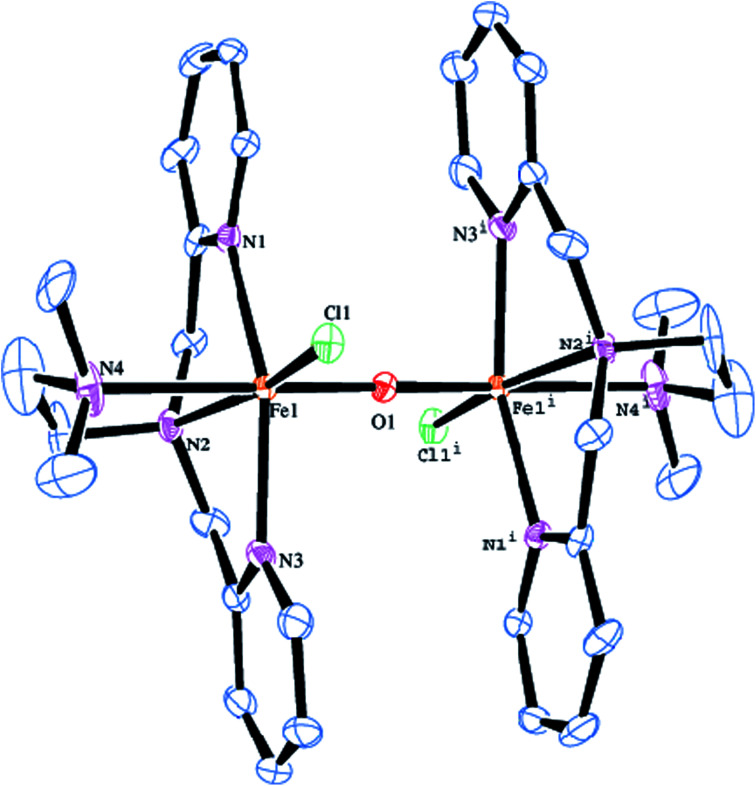
Molecular structure of [Fe_2_O(L2)_2_Cl_2_]^2+^2 (50% probability factor for the thermal ellipsoid). Hydrogen atoms have been omitted for clarity.

The molecular structure of [Fe_2_(μ-O)(L2)_2_Cl_2_]^2+^2 is shown in Fig. 1, together with the atom numbering scheme and the selected bond lengths and bond angles are collected in Table 2. The molecule contains an inversion centre and each iron atom in the diiron(iii) core possesses a distorted octahedral coordination geometry constituted by all the four amine nitrogen atoms of the ligand, the oxygen atom of μ-oxo bridge and a chloride ion. The Fe–N_py_ bond length (2.139(3), 2.130(3) Å) are shorter than the Fe–N_amine_ bond length (2.218(3), 2.371(4) Å), obviously due to the sp^2^ and sp^3^ hybridizations respectively of the pyridyl and tertiary amine nitrogen atoms. The terminal Fe–N_amine_ bond (2.371(4) Å) is longer than the central Fe–N_amine_ bond (2.218(3) Å) due to the trans effect exerted by the strongly coordinated μ-oxo bridge (Fe–O, 1.7957(5) Å) rather than chloride ion (2.2999(11) Å). Further, the coordination geometry of the complex cation is very similar to that for the already reported (μ-oxo)diiron(iii) core structure in the literature.^47,53,81^ The Fe–(μ-O) bond length of 1.7957(5) Å (Fe1–O1), fall in the range found for other μ-oxo diiron complexes (1.75–1.80 Å) and very close to methemerythrin (∼1.78 Å) and Fe_2_OCl_6_^2−^. The Fe–O–Fe bond angle of 180.0° suggests that the (μ-oxo)diiron(iii) core has a linear structure. The Fe⋯Fe distance is 3.541 Å, which is in the range found for the already reported complexes with Fe–O–Fe core (3.35–3.55 Å).^47,53,58,62^

**Fig. 2:**
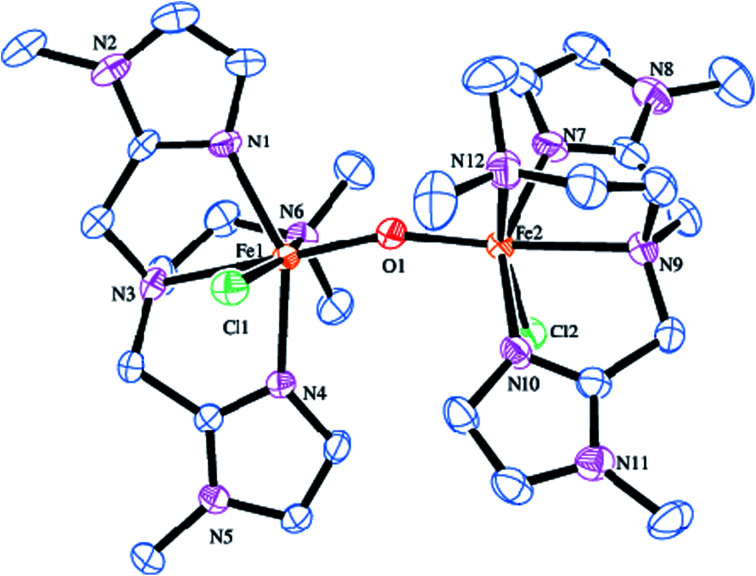
Molecular structure of [Fe_2_O(L5)_2_Cl_2_]^2+^5 (35% probability factor for the thermal ellipsoid). Hydrogen atoms have been omitted for clarity.

The molecular structure of [Fe_2_(μ-O)(L5)_2_Cl_2_]^2+^5 is shown in Fig. 2, together with the atom numbering scheme and the selected bond lengths and bond angles are collected in Table 2. The molecule contains no inversion centre and each iron atom in 5 possesses a distorted octahedral coordination geometry with slight difference in bond lengths and bond angles and is similar to that of 2. The Fe–N_im_ bond lengths (2.099(4), 2.095(4) Å) are shorter than the Fe–N_amine_ bond lengths (2.367(4), 2.277(4) Å), obviously due to the sp^2^ and sp^3^ hybridizations respectively of the imidazolyl and tertiary amine nitrogen atoms. The Fe–N_im_ bond lengths (2.099(4), 2.095(4) Å) in 5 are shorter than the Fe–N_py_ bond lengths (2.139(3), 2.130(3) Å) in 2, revealing that the imidazolyl nitrogen is coordinated more strongly than the pyridyl nitrogen. The terminal Fe–N_amine_ bond (2.277(4) Å) is shorter than the central Fe–N_amine_ bond (2.367(4) Å) due to the trans effect exerted by the strongly coordinated μ-oxo-bridge (Fe–O, 1.791(4) Å) and weak coordination of the chloride ion (2.4108(15) Å). In 5, the central amine nitrogen is coordinated trans to the μ-oxo-bridge rendering the central amine nitrogen to coordinate weakly with the iron(iii) center. This is in contrast to 2 where the central amine nitrogen is coordinated more strongly than the terminal nitrogen atom bound trans to the μ-oxo-bridge. The chloride ion is also coordinated more weakly in 5 (2.4108(15) Å) than in 2 (2.2999(11) Å). Interestingly, upon replacing the pyridyl nitrogen donor as in 2 by the imidazolyl nitrogen donor as in 5, the Fe–O–Fe bond angle decreases to 161.1(2)° and becomes bent, which may be due to the strong coordination of the imidazolyl nitrogen atoms and weak coordination of the central amine nitrogen atoms. In both 2 and 5, the N–Fe–N, N–Fe–O, N–Fe–Cl, O–Fe–Cl (78.64–101.52°) and N–Fe–N, N–Fe–O, N–Fe–Cl (154.14–165.13°) bond angles deviate from the ideal octahedral angles of 90° and 180° respectively, revealing the presence of significant distortion in the diiron(iii) coordination geometry.^82^

### Electronic absorption spectral studies

**Fig. 3:**
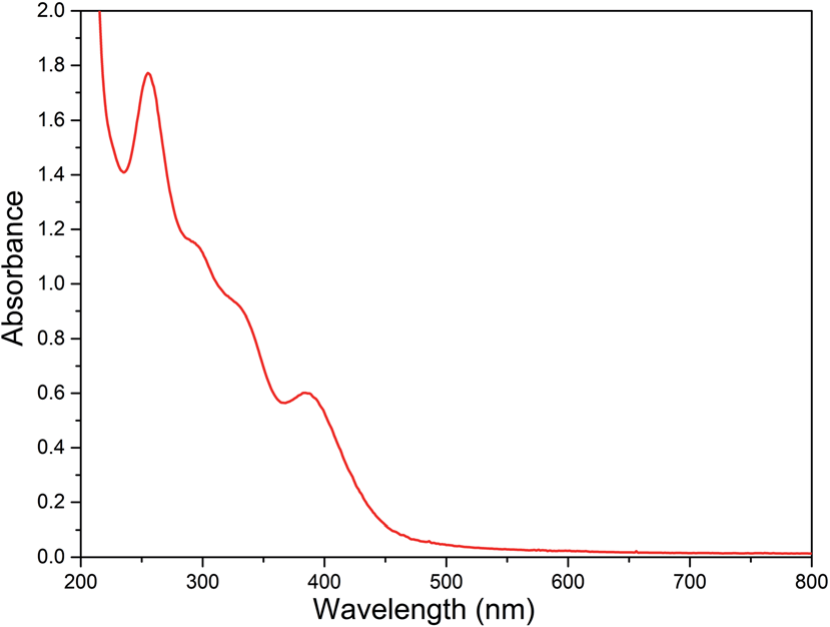
Electronic absorption spectra of [Fe_2_O(L2)_2_Cl_2_]Cl_2_2 (8.88 × 10^−5^ M) in MeOH : ACN mixture at 25.0 °C.

The electronic spectral data of all the diiron(iii) complexes are summarized in Table 3 and the typical electronic absorption spectrum of 2 is shown in Fig. 3. In MeOH : ACN (1 : 3 v/v) solvent mixture, all the present diiron(iii) complexes exhibit two absorption bands in the ranges 250–285 and 370–400 nm. The lower energy band in the range 370–400 nm is assigned to weak μ-oxo-to-Fe(iii) ligand to metal charge transfer transition (LMCT). The higher energy band in the range 250–285 nm is assigned to π–π* transition in the ligand moiety. The spectral properties of all the diiron(iii) complexes are very similar to those found for all of the previously reported diiron(iii) complexes of the same type, revealing the similarities in the structures of these complexes.^47,58^ Also, there is no significant difference in spectral behavior of the diiron(iii) complexes and mononuclear iron(iii) complexes of the same ligand has been observed. It has been previously reported that the μ-oxo-to-Fe(iii) CT transition for all the diiron(iii) complexes has been found to be blue-shifted when the Fe–O–Fe bond angle of diiron(iii) core changes.^83^ Thus, for the (μ-oxo)diiron(iii) complexes, upon increasing the Fe–O–Fe angle, the 400–500 nm absorption bands corresponding to μ-oxo-to-Fe(iii) CT transition are blue shifted with a decrease in molar absorptivity. Solomon *et al.* have suggested that these bands, which are principally weak oxo-to-iron(iii) ligand-to-metal charge transfer (LMCT) transitions, undergo blue-shift upon increasing the Fe–O–Fe bond angle towards 180° because the bonding between the iron atoms and the oxo atom becomes stronger when the Fe–O–Fe bond becomes linear.^84^ We have also observed the same blue shift when the bond angle tends to become 180°.

**Table 3:** UV-visible spectral data and electrochemical data of the diiron(iii) complexes in ACN/MeOH mixture at 25 °C^a^

Complex	*λ*_max_/nm (*ε*/M^−1^ cm^−1^)	*E*_p,c_ (CV) (V)	*E*_1/2_ (DPV) (V)	Redox process
[Fe_2_(O)(L1)_2_Cl_2_]^2+^	380 (7840)	−0.585	−0.489	Fe^III^Fe^III^ → Fe^II^Fe^III^
	326 (s, 8671)			
	254 (21 960)			
[Fe_2_(O)(L2)_2_Cl_2_]^2+^	385 (6770)	−0.545	−0.511	Fe^III^Fe^III^ → Fe^II^Fe^III^
	335 (s, 10 240)			
	255 (19 930)			
[Fe_2_(O)(L3)_2_Cl_2_]^2+^	382 (6250)	−0.504	−0.476	Fe^III^Fe^III^ → Fe^II^Fe^III^
	332 (s, 9630)			
	254 (18 830)			
[Fe_2_(O)(L4)_2_Cl_2_]^2+^	390 (5170)	−0.572	−0.538	Fe^III^Fe^III^ → Fe^II^Fe^III^
	330 (s, 7760)			
	255 (16 990)			
[Fe_2_(O)(L5)_2_Cl_2_]^2+^	362 (8300)	−0.623	−0.585	Fe^III^Fe^III^ → Fe^II^Fe^III^
	283 (15 080)			
[Fe_2_(O)(L6)_2_Cl_2_]^2+^	396 (5840)	−0.481	−0.442	Fe^III^Fe^III^ → Fe^II^Fe^III^
	335 (s, 9820)			
	260 (20 100)			

a Potential measured *vs.* Ag/AgNO_3_ (0.001 M, 0.1 M TBAP); add 0.544 V to convert to NHE.

### Electrochemical properties

**Fig. 4:**
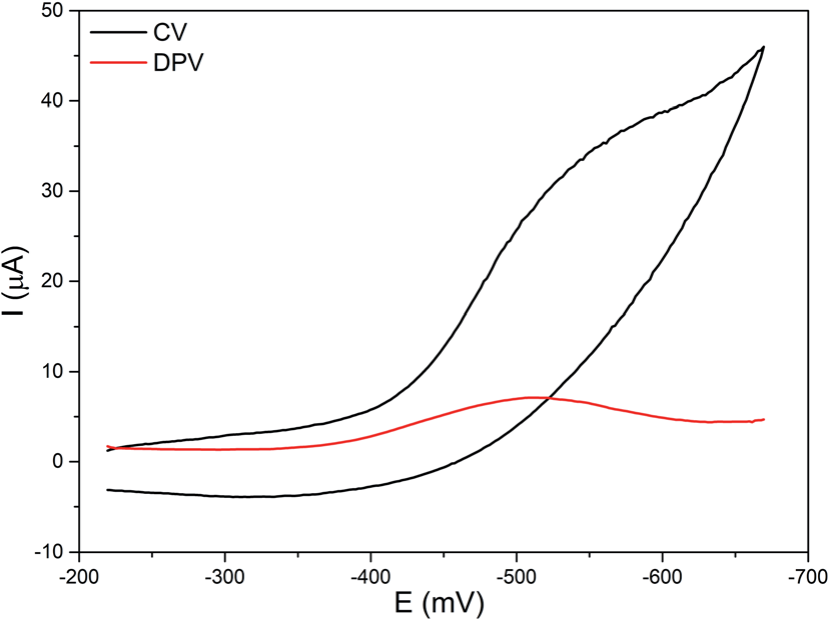
Cyclic (CV) and differential pulse voltammogram (DPV) of 2 in methanol/acetonitrile mixture at 25 °C. Supporting electrolyte: 0.1 m TBAP. Scan rate: for CV 50 mV s^−1^, for DPV 5 mV s^−1^.

The electrochemical properties of the diiron(iii) complexes were investigated in methanol : acetonitrile solvent mixture by employing cyclic (CV) and differential pulse voltammetry (DPV) on a stationary platinum electrode. All of the complexes show a cathodic reduction wave in the range −0.48 to −0.62 mV, but not any coupled oxidation wave in the CV (Fig. 4).Fe(iii)–O–Fe(iii) + e^−^ → Fe(ii)–O–Fe(iii)

The *E*_1/2_ values of the Fe^III^/Fe^II^ redox couples (−0.44 to −0.58 V, Table 3) fall in the range observed for similar type of oxo-bridged diiron(iii) complexes. They are highly negative mainly due to the strong coordination of the bridging oxo-group and chloride ions and follow the trend 1 < 2 < 3 > 4 > 5 < 6. On replacing one of the pyridyl nitrogen donors in 1 by –NMe_2_ group to obtain 2, the Fe^III^/Fe^II^ redox potential is shifted to less negative values due to the weaker coordination of the sterically hindered –NMe_2_ group to iron(iii) center. A similar shift in the Fe^III^/Fe^II^ redox potential from less negative region to more negative region is observed upon replacing both the pyridyl nitrogen donors in 2 by 6-methylpyridyl donor to obtain 3, revealing that the methyl group on the pyridyl ring makes the pyridyl nitrogen to coordinate weakly with the iron(iii) center. Whereas on replacing one of the pyridyl nitrogens in 2 by *N*-Me-imidazolyl donor to obtain 4, the Fe^III^/Fe^II^ redox potential is shifted to more negative values due to the stronger coordination of the electron-releasing *N*-Me-imidazole (p*K*_a_: pyH^+^, 5.2, MeImH^+^, 7.0) nitrogen donor and hence its stronger coordination as in 2. The Fe^III^/Fe^II^ redox potential is further shifted to more negative value upon replacing both the pyridyl donor in 2 by *N*-Me-imidazolyl donor to obtain 5, which is consistent with the Fe–N_im_ bond length observed for 5 being shorter than the Fe–N_py_ bond length for 2 (*cf.* above). But, the Fe^III^/Fe^II^ redox potential is shifted to more positive value upon replacing both the pyridyl donor in 2 by the quinolyl donor to obtain 6 due to the coordination of the bulky quinolyl group weaker than the pyridyl donor. All the above observations reveal that the introduction of strong donor, leading to the shift in Fe^III^/Fe^II^ redox potential to more negative values, renders the FeN_4_OCl coordination sphere more compact stabilizing iron(iii) oxidation state. Whereas the FeN_4_OCl coordination sphere of complexes with quinolyl or pyridyl nitrogen donors is less compact, as evident from their less negative Fe^III^/Fe^II^ redox potential. Also, both electronic as well as steric effects play a major role in determining the Lewis acidity of the diiron(iii) center and the redox potential is well tuned upon varying the ligand donor functionalities.

### Reaction of diiron(iii) complexes with *m*-CPBA

**Fig. 5:**
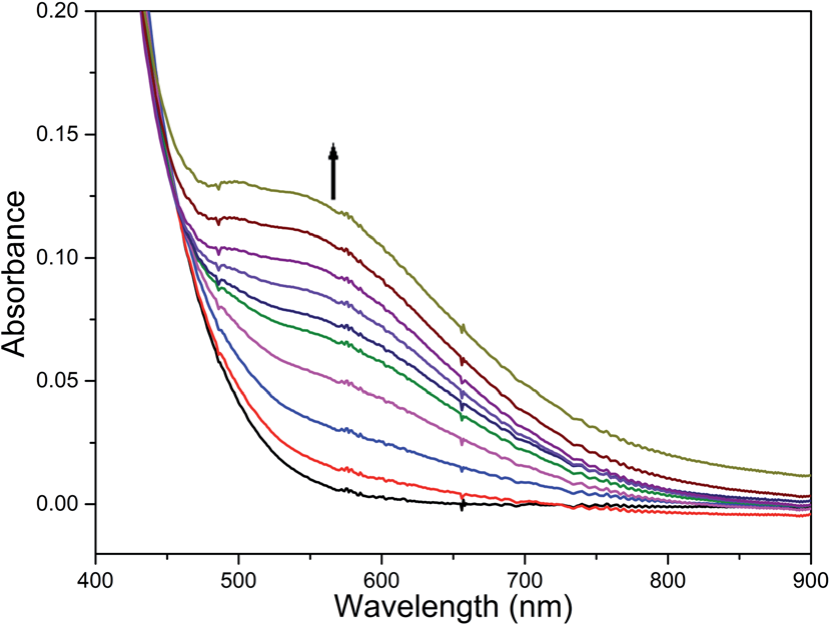
Reaction of complex 2 with AgClO_4_ and *m*-CPBA (1 equiv.) and triethylamine (1 equiv.) followed by UV-visible spectroscopy at room temperature.

**Scheme 3:**
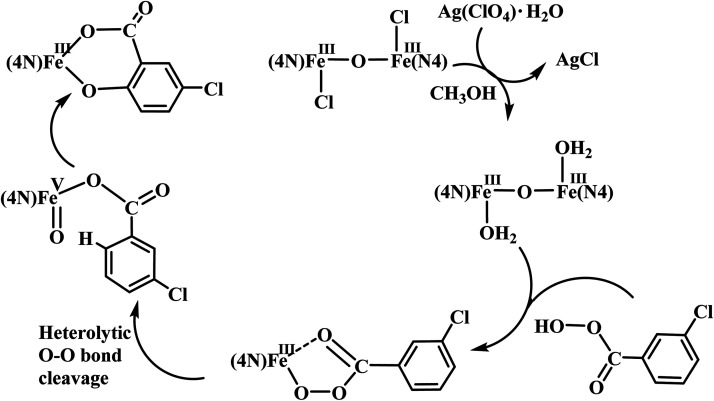
Proposed mechanism of intramolecular arene hydroxylation.

**Fig. 6:**
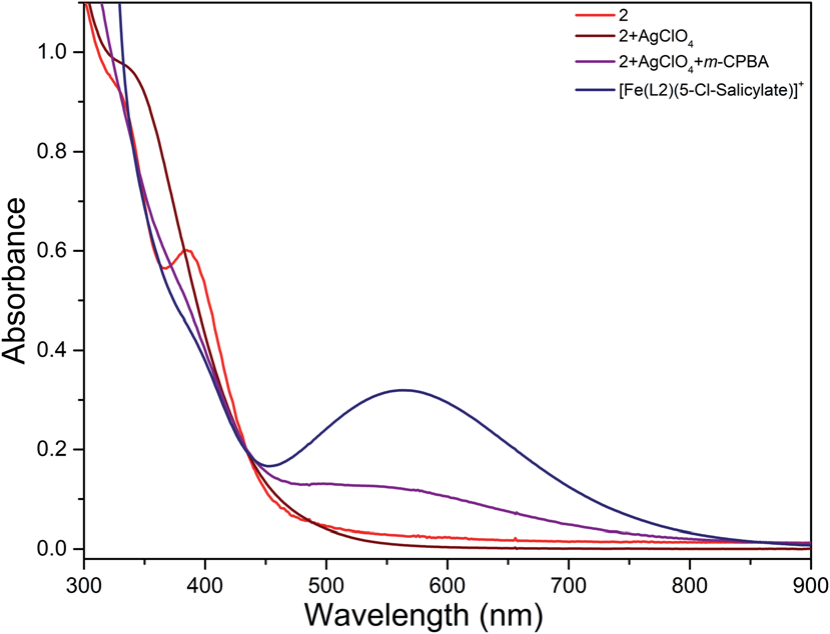
Electronic absorption spectra of 2 (8.88 × 10^−5^ M) before and after treatment with silver perchlorate and *m*-CPBA and [Fe(L2)(5-Cl-salicylate)]^+^ (8.88 × 10^−5^ M) in MeOH : ACN mixture at 25.0 °C.

The reaction of diiron(iii) complex 2 with *m*-CPBA in methanol at room temperature was investigated using UV-visible spectroscopy. No appreciable changes were observed when 2 was treated with *m*-CPBA, revealing that the strong coordination of chloride ion with iron(iii) center, renders the complex less reactive towards the oxidant. When the diiron(iii) complex 2 was treated with silver perchlorate monohydrate to remove the coordinated chloride ions as silver chloride by centrifugation. The electronic absorption spectrum of the supernatant solution is found to be similar to that of the diiron(iii) complexes with slight shift in wavelengths towards higher energy region. The reaction of supernatant liquid with *m*-CPBA produced a pink colored species showing a new absorption band around 565 nm (Fig. 5). ESI-MS analysis of the pink solution shows a prominent peak cluster at *m*/*z* value of 495.96, corresponding to the presence of the intramolecular oxo-transferred species [(L2)Fe(5-Cl-salicylate)]^+^. When the pink solution was treated with small amount of con. HCl and extracted with dichloromethane, the GC-MS analysis of the extract shows the formation of 5-chlorosalicylic acid, revealing that upon binding with the iron(iii) center *m*-CPBA undergoes intramolecular oxo transfer to the phenyl ring, that is, self-hydroxylation of *m*-CPBA. When iron(iii) perchlorate was treated with L2, 5-chlorosalicylic acid and triethylamine in acetonitrile, the complex [(L2)Fe(5-Cl-salicylate)]^+^ was formed, as diagnosed by an absorption band around 565 nm. This confirmed that the new species formed upon reaction of 2 with *m*-CPBA corresponds to [(L2)Fe(5-Cl-salicylate)]^+^. Interestingly, the treatment of mononuclear chlorido complex [Fe(L2)Cl_2_]^+^ of the same ligand L2 does not involve in intramolecular oxo-transfer of *m*-CPBA, but the perchlorate complexes take part in the intramolecular oxo transfer reaction, revealing that at least two vacant sites on the complex species are needed for self-hydroxylation of *m*-CPBA, as reported earlier (Fig. 6).^85^ So, it is clear that upon treatment of the diiron(iii) complexes with silver perchlorate the dimeric core is broken to form monomeric solvent coordinated species, which then takes part in the intramolecular oxo transfer. Nam *et al.* have previously observed the self-hydroxylation of *m*-CPBA upon treating the iron(ii) complex [Fe(TPA)(NCCH_3_)_2_]^2+^ with *m*-CPBA and proposed the involvement of high-valent (4N)Fe^V^=O species in this reaction.^86^ Whereas, Rybak-Akimova *et al.* observed the *ortho*-hydroxylation of benzoic acids upon treating the iron(ii) complex [Fe(BPMEN)(NCCH_3_)_2_]^2+^, where BPMEN is bis(2-pyridylmethyl)ethylenediamine, with benzoic acid and H_2_O_2_.^87,88^ Here we observe that the monomeric iron(iii) complex species formed from diiron(iii) complexes carries out the intramolecular oxo transfer reaction. It is proposed that the oxidant *m*-CPBA binds to the monomeric iron(iii) center in a bidentate fashion and undergoes heterolytic O–O bond cleavage leading to the formation of (4N)(5-Cl-benzoate)Fe^V^=O species. The latter abstracts a proton from the phenyl ring of iron(iii)-bound *m*-CPBA and rebound to the same phenyl ring to form 5-chlorosalicylic acid coordinated to the iron(iii) center (Scheme 3).

### Catalytic oxidations of alkanes by diiron(iii) complexes

The experimental conditions and the results of catalytic oxidation of alkanes into alcohols for all the diiron(iii) complexes 1–6 are summarized in Tables 4 and 5. The conversion of alkanes into hydroxylated products was quantified by employing gas chromatographic analysis involving authentic samples and an internal standard. The catalytic ability of the diiron(iii) complexes towards oxidation of alkanes like cyclohexane and adamantane was explored by using *m-*CPBA, H_2_O_2_ and *t*-BuOOH as oxidants in CH_2_Cl_2_ : CH_3_CN solvent mixture (3 : 1 v/v) at room temperature. Also, it was found that H_2_O_2_ and *t*-BuOOH were not effective oxidants for hydroxylation of alkanes. Control reactions performed in the absence of the diiron(iii) complexes with *m*-CPBA as oxidant yielded only very small amounts of the oxidized products for all the substrates (cyclohexane, 3 TON; adamantane, 5 TON). In the presence of the complexes, the oxidation of cyclohexane proceeds to give cyclohexanol as the major product along with cyclohexanone, chlorocyclohexane and ε-caprolactone as the minor products. ε-Caprolactone is the over oxidized product of oxidation of cyclohexanone by the excess or unreacted *m*-CPBA. The small amount of chlorocyclohexane detected for all the complexes is formed by the oxidative ligand transfer (OLT) pathway under catalytic conditions. Thus, all the present diiron(iii) complexes act as robust catalysts towards the oxidation of alkanes to alcohols.

**Table 4:** Products of oxidation of cyclohexane catalyzed^a^ by diiron(iii) complexes

Complex	Cyclohexane (TON)			Total TON^c^	A/K^d^	Yield^e^
	-ol^b^	-one^b^	ε-Caprolactone			
1	362	57	12	431	5.2	61.5
2	430	48	16	494	6.7	70.5
3	390	52	14	456	5.9	65.1
4	448	51	12	513	7.1	73.2
5	370	58	23	451	4.5	64.4
6	332	43	15	390	5.7	55.7

a Reaction conditions: catalyst (1 × 10^−3^ mmol dm^−3^), substrate (3 mol dm^−3^), oxidant (0.7 mol dm^−3^) in DCM : ACN solvent mixture (9 : 1 v/v).

b -ol = cyclohexanol and -one = cyclohexanone.

c Total TON = no. of mmol of product/no. of mmol of catalyst.

d A/K = TON of -ol/(TON of -one + TON of ε-caprolactone).

e Yield based on the oxidant.

**Table 5:** Products of oxidation of adamantane catalyzed^a^ by diiron(iii) complexes

Complex	Adamantane (TON)			Total TON^c^	Selectivity^d^	Yield^e^
	1-adol^b^	2-adol^b^	2-adone^b^		3°/2°	
1	241	53	15	310	10.6	51.6
2	313	43	38	454	11.6	65.1
3	278	46	31	355	10.8	59.1
4	260	60	25	345	09.1	57.5
5	272	28	16	316	18.5	52.6
6	256	48	21	325	11.1	54.1

a Reaction conditions: catalyst (1 × 10^−3^ mmol dm^−3^), substrate (1 mol dm^−3^), oxidant (0.5 mol dm^−3^) in DCM : ACN solvent mixture (4 : 1 v/v).

b 1-adol = 1-adamantanol, 2-adol = 2-adamantanol and 2-adone = 2-adamantanone.

c TON = no. of mmol of product/no. of mmol of catalyst.

d 3°/2° = (TON of 1-adol × 3)/(TON of 2-adol + TON of 2-adone).

e Yield based on the oxidant.

Complex 1 catalyzes the oxidation of cyclohexane to 362 TON of cyclohexanol (A) and 57 TON of cyclohexanone (K) and 12 TON of ε-caprolactone (A/K, 5.2) with 60% conversion of oxidant to oxidized products. The observed A/K value for cyclohexane oxidation suggests the involvement of a high-valent iron–oxo species rather than a freely diffusing radical species (A/K ≈ 1 for radical reaction) in the catalytic reaction. In contrast to the high TON observed when *m*-CPBA is used as oxidant for hydroxylation of alkanes, the complex 1 shows a very low TON when H_2_O_2_ or *t*-BuOOH is used as the oxidant. Upon replacing one of the pyridyl donors in 1 by a weakly coordinating –NMe_2_ group to obtain 2, the catalytic oxidation of cyclohexane occurs to provide 430 TON of cyclohexanol, 48 TON of cyclohexanone and 16 TON of ε-caprolactone. This may be due to the weak coordination of the –NMe_2_ group rather than the pyridyl donor as revealed from the crystal structure makes the bridging oxo-group leading to the decrease in Lewis acidic character of the iron(iii) center, which may stabilize the high-valent iron–oxo species involved in the catalytic reaction. Previously it was reported that the stability of the high-valent iron–oxo species generated from certain mononuclear iron(ii) complexes has been correlated with the number of pyridine donors present in the primary ligand. So, the present diiron(iii) complex is also expected to stabilize the high-valent iron–oxo species so that they can act as efficient turn over catalyst for alkane hydroxylation. Thus the behavior of 2 towards alkane substrates can be compared with several non-heme iron catalysts: (a) the Gif family of catalysts, which afford mainly ketone products;^89^ (b) catalysts with A/K ≈ 1 such as [Fe_2_O(OAc)_2_(bpy)_2_]Cl_2_, [Fe_2_O(OAc)_2_(tmima)_2_](ClO_4_)_3_, [Fe(pma)](ClO_4_)_2_ and [Fe_2_O(bpy)_4_(H_2_O)_2_](ClO_4_)_4_;^90–92^ and (c) catalyst with large A/K ratios such as [Fe(bpmen)(CH_3_CN)_2_](ClO_4_)_2_ (A/K = 9.5), [Fe(tpa)(CH_3_CN)_2_](ClO_4_)_2_ (A/K = 12.0) and [Fe(N4Py)(CH_3_CN)](ClO_4_)_2_ (A/K = 7.9).^76,93,94^ Thus the A/K ratio (12.2) found for 2 corresponds most closely to those associated with the catalyst group c. Upon replacing both the pyridyl nitrogen donor in 2 by 6-methylpyridyl donor to obtain 3, the catalytic oxidation of cyclohexane occurs to yield 390 TON of cyclohexanol, 52 TON of cyclohexanone and 14 TON of ε-caprolactone. Upon replacing the pyridyl donors in 1 by the 6-methylpyridyl donor both the catalytic activity and selectivity decrease due to the weaker coordination of the later one. Interestingly, upon replacing one of the pyridyl donors in 2 by *N*-Me-imidazolyl nitrogen donor to obtain 4, cyclohexane is oxidized to 450 TON of cyclohexanol, 51 TON of cyclohexanone and 12 TON of ε-caprolactone. Upon introduction of the strongly coordinating *N*-Me-imidazolyl group both the catalytic activity and selectivity increased. But, upon replacing both the pyridyl donors in 2 by *N*-Me-imidazolyl donor to obtain 5, the catalytic oxidation of cyclohexane proceeds to give 370 TON of cyclohexanol, 58 TON of cyclohexanone and 23 TON of ε-caprolactone. Upon introduction of two *N*-Me-imidazolyl nitrogen donor it is expected that the total TON and selectivity increase; however, we observe both the total TON and selectivity to decrease. Upon replacing both the pyridyl nitrogen donors in 2 by quinolyl nitrogen donors to obtain 6, the catalytic oxidation of cyclohexane occurs to give 332 TON of cyclohexanol, 43 TON of cyclohexanone and 15 TON of ε-caprolactone.

### Adamantane oxidation

The catalytic activity of all the diiron(iii) complexes 1–6 towards oxidation of adamantane has been also explored and the results are summarized in Table 5. All the complexes catalyze the oxidation of adamantane efficiently to give 1-adamantanol and 2-adamantanol as the major products along with 2-adamantanone as the minor product. Complex 1 catalyzes the oxidation of adamantane to give 241 TON of 1-adamantanol, 53 TON of 2-adamantanol and 15 TON of 2-adamantanone (total TON, 310) with a good selectivity (3°/2°, 10.6). However, 2 catalyzes adamantane oxidation to give 313 TON of 1-adamantanol, 43 TON of 2-adamantanol and 38 TON of 2-adamantanone with increase in catalytic activity (total TON, 454). This trend is the same as that observed for cyclohexane oxidation, revealing that the electron-releasing nature of the donor atom play a significant role on the catalytic reaction of the diiron(iii) center as well as formation and stabilization of the high-valent iron–oxo intermediate. Complex 3 also catalyses adamantane oxidation to give 355 TON of oxidized products, which is higher than that observed for 1. Whereas, the introduction of strongly σ-bonding *N*-Me-imidazolyl donor the complexes 4 and 5 catalyze with lower TON compared to complexes 1–3. However, complex 5 catalyzes oxidation of adamantane with very high selectivity (3°/2°, 18.5), which may be due to the stabilization of high-valent iron–oxo species. The high 3°/2° selectivity observed indicates the involvement of high-valent iron–oxo species in adamantane oxidation also.

**Scheme 4:**
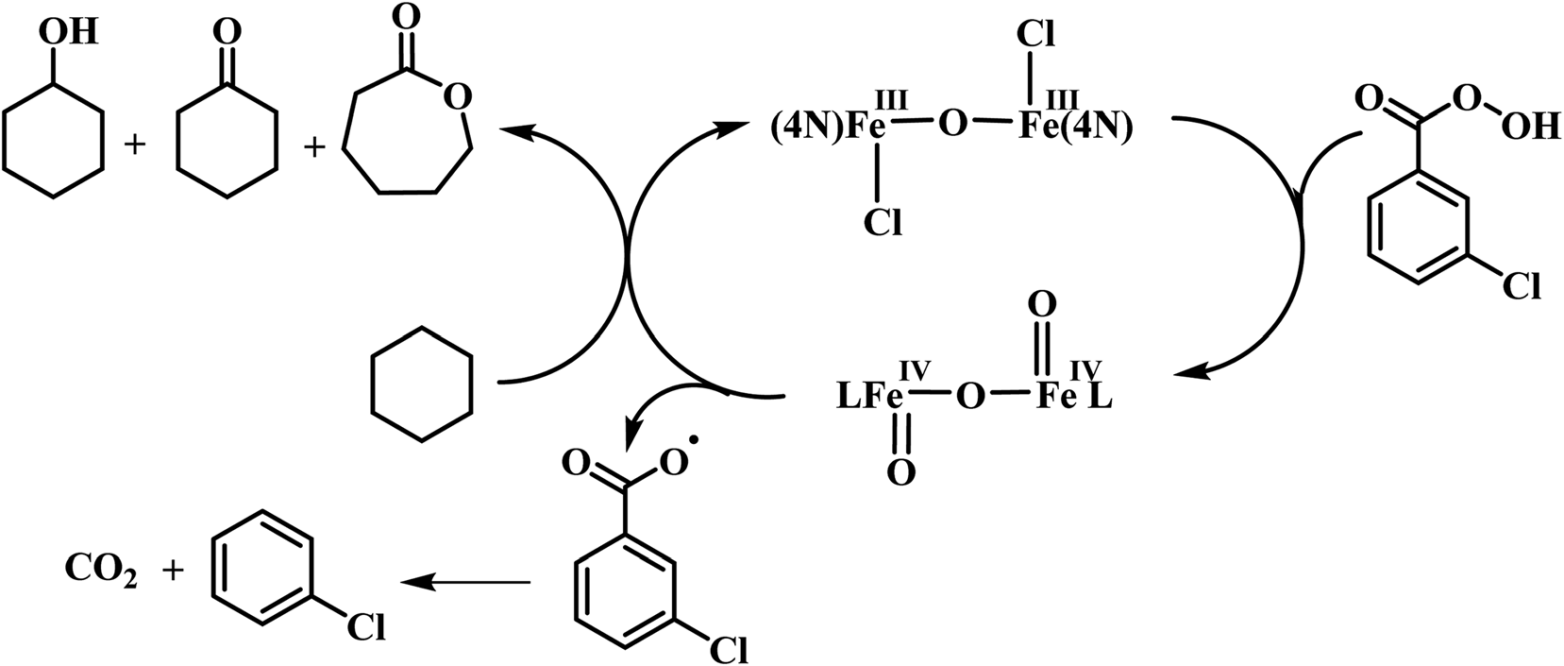
Proposed mechanism for alkane hydroxylation.

Interestingly, all the present diiron(iii) complexes show higher selectivity in the hydroxylation of cyclohexane (A/K, 5–7; Table 4) and adamantane (3°/2°, 9–18; Table 5), signifying the involvement of metal-based oxidants rather than non-selective freely diffusing radical species in the alkane hydroxylation. Under nitrogen atmosphere, almost the same type of reactivity pattern was observed, revealing that the cyclohexylperoxide species is not involved in the catalytic reaction. This observation also strongly supports the involvement of metal-based oxidants.^89,95,96^ We propose that the *m*-CPBA binds with diiron(iii) center by replacing a chloride ion to form the adduct [Fe_2_(O)(L)_2_Cl(OOCOC_6_H_4_Cl)]^3+^, which undergoes O–O bond homolysis leading to the formation of the Fe^IV^=O reactive intermediate species and *m*-chlorobenzoate radical or O–O bond heterolysis leading to the formation of Fe^V^=O species and *m*-chlorobenzoic acid (Scheme 4). In the former case, the Fe^IV^=O species formed would then be involved in hydroxylation of alkanes while *m*-chlorobenzoate radical undergoes decarboxylation to form chlorobenzene in more than 50% yield. The observation of chlorobenzene supports the involvement of the Fe^IV^=O intermediate species and favors the O–O bond homolysis during catalysis. Also, the *m*-chlorobenzoate radical might be a very minor reaction intermediate because it readily undergoes decarboxylation rather than abstracting hydrogen from alkanes under room temperature.

## Conclusions

A few non-heme μ-oxo-bridged diiron(iii) complexes of tripodal 4N ligands have been isolated and characterized by spectral and electrochemical methods. In the X-ray crystal structures of the molecules 2 and 5, both the iron(iii) centers possess a distorted octahedral coordination geometry. All the diiron(iii) complexes catalyze the hydroxylation of cyclohexane and adamantane efficiently with good selectively in the presence of *m*-CPBA as oxidant. The observed selectivity for cyclohexane (A/K; 5–7) and adamantane (3°/2°; 9–18) suggest the involvement of high-valent iron–oxo species rather than freely diffusing radicals in the catalytic reaction. Interestingly, 4 oxidizes cyclohexane (A/K, 7) very efficiently up to 513 TON while 5 oxidizes adamantane with good selectivity (3°/2°, 18) in the presence of *m*-CPBA within one hour. The stereoelectronic effects of ligand donors play a vital role in determining the catalytic efficiency of the diiron(iii) complexes towards hydroxylation of alkanes. Interestingly, the incorporation of the strongly coordinating *N*-methylimidazole donor renders the complex to act as efficient catalyst by stabilizing the high-valent iron–oxo intermediate species whereas the incorporation of weakly coordinating quinolyl donor makes the complex to act as a relatively poor catalyst by destabilizing the high-valent iron–oxo intermediate species.

## Conflicts of interest

There are no conflicts to declare.

## Supplementary Material

RA-011-D1RA03135J-s001

RA-011-D1RA03135J-s002

RA-011-D1RA03135J-s003
